# Genetic diversity and demographic history of introduced sika deer on the Delmarva Peninsula

**DOI:** 10.1002/ece3.5655

**Published:** 2019-09-27

**Authors:** David M. Kalb, Deborah A. Delaney, Randy W. DeYoung, Jacob L. Bowman

**Affiliations:** ^1^ Virginia Department of Game and Inland Fisheries Marion VA USA; ^2^ Department of Entomology and Wildlife Ecology University of Delaware Newark DE USA; ^3^ Caesar Kleberg Wildlife Research Institute Texas A&M University‐Kingsville Kingsville TX USA

**Keywords:** approximate Bayesian computation, *Cervus nippon yakushimae*, founder effect, genetic diversity, invasive species, non‐native species, serial founders

## Abstract

The introduction of non‐native species can have long‐term effects on native plant and animal communities. Introduced populations are occasionally not well understood and offer opportunities to evaluate changes in genetic structure through time and major population changes such as bottleneck and or founder events. Invasive species can often evolve rapidly in new and novel environments, which could be essential to their long‐term success. Sika deer are native to East Asia, and their introduction and establishment to the Delmarva Peninsula, USA, is poorly documented, but probably involved ≥1 founder and/or bottleneck events. We quantified neutral genetic diversity in the introduced population and compared genetic differentiation and diversity to the presumed source population from Yakushima Island, Japan, and a captive population of sika deer in Harrington, Delaware, USA. Based on the data from 10 microsatellite DNA loci, we observed reduced genetic variation attributable to founder events, support for historic hybridization events, and evidence that the population did originate from Yakushima Island stocks. Estimates of population structure through Bayesian clustering and demographic history derived from approximate Bayesian computation (ABC), were consistent with the hypothesized founder history of the introduced population in both timing and effective population size (approximately five effective breeding individuals, an estimated 36 generations ago). Our ABC results further supported a single introduction into the wild happening before sika deer spread throughout the Delmarva. We conclude that free‐ranging sika deer on Delmarva are descended from ca. five individuals introduced about 100 years ago from captive stocks of deer maintained in the United Kingdom. Free‐ranging sika deer on Delmarva have lost neutral diversity due to founder and bottleneck events, yet populations have expanded in recent decades and show no evidence of abnormalities associated with inbreeding. We suggest management practices including increasing harvest areas and specifically managing sika deer outside of Maryland.

## Graphical abstract

The introduction of sika deer on to Maryland's Eastern Shore has resulted in significant bottlenecks in neutral genetic diversity of the 12,000 deer now in the region. This paper summarized the cause of these bottlenecks and the potential implications to the community and future management of the game animal.

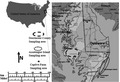

## 1 INTRODUCTION

The introduction of non‐native species may have long‐lasting ecological impacts to native communities (Sakai et al., 2001). While non‐natives may provide some benefits, many also compete with or harm native species (Kalb, Bowman, & DeYoung, 2018). The factors that influence success of introduced populations are not well understood, and it is difficult to predict which introductions will become invasive (Sakai et al., 2001). The genetic diversity and structure of introduced populations may hold key insights into their ability to expand and adapt to novel environments (Sakai et al., 2001). Although non‐natives often display reduced genetic diversity resulting from founder effect and genetic drift, some populations are able to avoid negative consequences of low genetic diversity or inbreeding (Lee, 2002). This observation has been termed the genetic paradox of invasive species (Estoup et al., 2016; Frankham, 2005). The paradox is not always valid, as some non‐natives that would otherwise suffer from low diversity, avoid this and retain evolutionary potential, through multiple introductions or admixture of source stocks (Estoup et al., 2016; Kolbe et al., 2004). Furthermore, loss of diversity during bottleneck events may purge deleterious variation in some cases (Hedrick, 2001). Clearly, the success of non‐natives depends in part on the introduction and founding history (Lee, 2002; Rius & Darling, 2014). Therefore, documentation of the genetic stocks involved and their demographic history should be useful in management (Cameron, Bayne, & Coltman, 2008; Sakai et al., 2001) as well as understanding the evolutionary response of introduced species to a new environment (Estoup et al., 2016; Lee, 2002).

**Figure 1:**
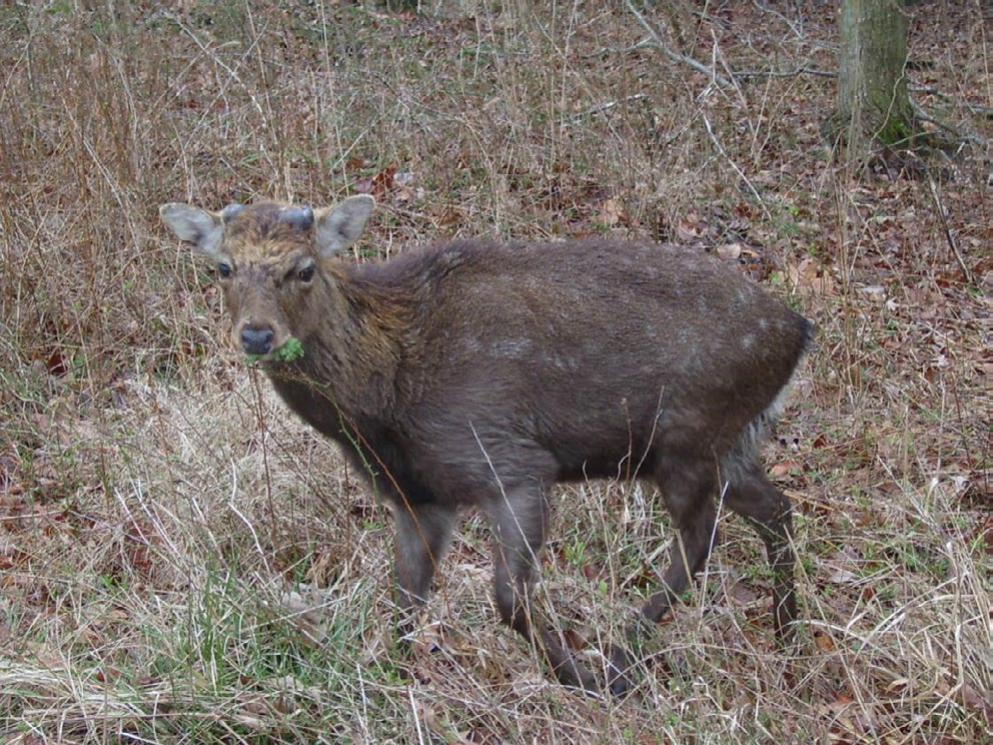
Adult male sika deer from Dorchester County Maryland, USA. Image is from early spring just as males begin growing antlers. A large adult male may reach 50 kg dressed weight

Native to East Asia, sika deer (*Cervus Nippon;* Figure 1) have been introduced worldwide for sport hunting or as alternative livestock (e.g., the velvet antler industry). Sika deer are considered invasive in many areas where they have been introduced due to their effects on and interactions with native wildlife (Kalb et al., 2018; Senn & Pemberton, 2009).

In the Japanese Islands, sika deer were geographically isolated from mainland Asia by a series of vicariant events concurrent with cycles of glaciation (Riss‐Würm) and changes in sea levels. These pervasive glacial events created small island areas with sika deer populations maintained by few individuals, which has resulted in isolated subspecies. Many of these populations have low neutral genetic diversity and are easily separated into clades based on mtDNA lineages (Tamate, 2009).

**Figure 2:**
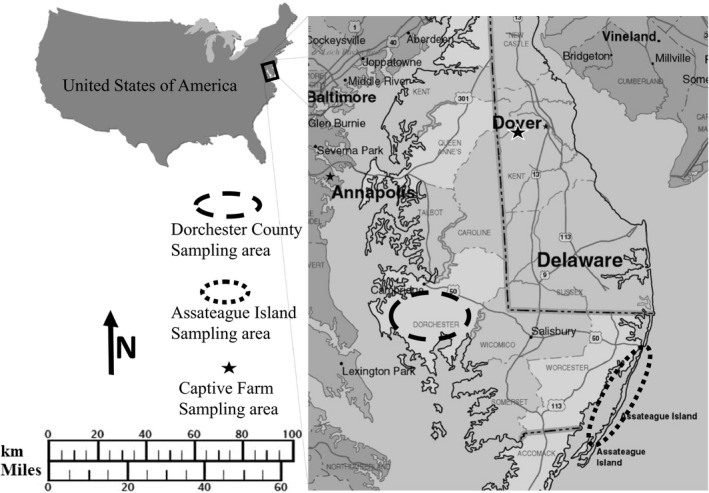
Map showing sample locations from Dorchester, Assateague, captive sika, and their general location on the Delmarva Peninsula relative to the Continental United States of America

The largest free‐ranging population of sika deer in North America occurs in the Mid‐Atlantic US on the Delmarva Peninsula (a region that encompasses Delaware and the eastern coasts of Maryland and Virginia; hereafter Delmarva; Figure 2) where they have been shown to compete with native white‐tailed deer (*Odocoileus virginianus*; Kalb et al., 2018). Records are sparse, but Japanese sika deer were apparently released to the Delmarva onto James Island in the Chesapeake Bay (2 km off the Delmarva mainland and part of Dorchester County, Maryland, USA) around 1916 as a single introduction of 4 or 5 individuals (Feldhamer & Demarais, 2009; Flyger, 1960; Kalb & Bowman, 2017; Kalb, Bowman, & Eyler, 2013).

The source population for free‐roaming sika deer on the Delmarva originated in Japan, but spent several generations at Woburn Abbey, England (Feldhamer & Demarais, 2009; Kalb & Bowman, 2017) prior to their arrival in the United States. While in England, the deer were held in captivity with other stocks of unknown origin and allowed to intermingle (Banwell, 1999). In 1924, roughly 8 years after their introduction to the USA, the population of sika deer in Dorchester County, MD, still primarily on James Island, was divided (unknown quantities), and some deer were moved to Assateague Island in Worchester County, MD, USA (Figure 2; Flyger, 1960). A severe wildfire in 1957 reduced the population of sika deer on James Island by nearly half (Flyger, 1959; Flyger & Bowers, 1958).

Sika deer are now locally abundant on the southern portion of the Delmarva. Despite their founding from so few individuals, sika deer on Delmarva demonstrate remarkable vigor and have experienced near exponential growth over the last century (Davidson & Crow, 1983; Kalb & Bowman, 2017). However, the degree of genetic variation within wild sika deer that may allow future adaptation is not well understood. White‐tailed deer have declined throughout portions of the Delmarva Peninsula resulting in areas where sika deer have become the primary cervid species. There is no evidence that it is possible for these two distinctly and evolutionarily different species (Polziehn & Strobeck, 2002) to hybridize with either sex‐species configuration and is unlikely to happen in vivo due to differences in breeding season behavior and timing.

The free‐ranging sika deer on the Delmarva are believed to be geographically isolated into two populations, one centering in and around Dorchester County MD, and the other around Assateague Island (Figure 2). In Dorchester MD, Sika deer are a valued, yet controversial game species. Annually there are thousands of harvests throughout several counties in Eastern Maryland with a large hunter interest due to the exotic nature of the animal and unique experience of the sika deer rut behavior. However, neighboring states of Virginia and Delaware, USA (as well as some National Wildlife Areas), do not want established sika deer populations.

Introduced populations often experience dramatic changes in effective population size and geographic range, with implications for the maintenance of genetic diversity (Dlugosch & Parker, 2008; Hunter & Gibbs, 2007). Historically, translocations for most species in general were not well monitored or detailed in records. Additionally, introductions by private citizens are often clandestine. Although we only have evidence of a single introduction through multiple founder events, an investigation of genetic diversity and demographic history will provide evidence to better understand sika deer on the Delmarva.

The possible effects of low genetic variability could impact long‐term management of this species. Low genetic diversity can have implications on the success of isolated populations, such as decreased fitness, and the limited ability of a population to adapt to changes in their environment (Baalsrud, 2011; Reed & Frankham, 2003). The introduced wild sika deer population offers a unique opportunity to investigate the effects of serial founder events on neutral genetic diversity (Sakai et al., 2001). A captive sika deer of a known different provenance (an unknown mixture of Manchurian sika deer [*C. n. mantchuricus*] and other stocks) on the Delmarva provide an excellent outgroup for genetic comparisons and are held within several high fenced enclosures in Harrington, DE. To the best of anyone's knowledge, no sika deer from this facility have ever escaped, and no sika deer from the wild have encountered the captive facility. The potential for admixture could aid in the invasive success of wild sika deer if any of the captive animals were to escape. Wild sika deer on the Delmarva are also isolated from other congeneric species (e.g., elk [*Cervus canadensis*]). If sika deer have been able to escape the captive site, genetic analysis should be able to confirm this and the degree to which they have hybridized with wild sika deer.

The overall goal of this study was to quantify and compare the genetic diversity in the wild sika deer on the Delmarva and use the data to inform what we currently understand about their introduction to the Delmarva. Therefore, we compared the genetic diversity in free‐ranging sika deer populations to the putative source population, as well as a population of captive sika deer on the Delmarva. We hope that an analysis of the neutral genetic fingerprint of all sika deer on the Delmarva will allow us to better understand how sika deer spread across the Peninsula through identification of unique markers within geographic populations. Because of the different demographic histories we expected to find greater allelic diversity and more private alleles within the captive sika deer and Yakushima, Japanese samples. We also modeled the demographic history of the wild sika deer on the Delmarva to provide additional information about timing of and size of founder events. Finally, we discuss mechanisms that may have led to the success of populations from small founder events.

## 2 MATERIALS AND METHODS

We collected tissue and/or fecal samples from sika deer from four geographically isolated locations; Assateague Island, MD, USA; Dorchester County, MD, USA; captive deer in Harrington, DE, USA (~80 km northeast of Dorchester County); and Yakushima Island, Japan, the presumed source population for wild sika deer on the Delmarva. Samples from Dorchester County and Assateague Island were collected from hunter‐harvested deer throughout the areas on both public and private lands. Both Dorchester and Assateague Island are comprised of salt marsh and mixed woody wetland habitats that hold sika deer in varying numbers.

We collected a 3 cm cube of liver or muscle tissue samples from 55 sika deer harvested throughout Dorchester County, and 30 sika deer harvested on Assateague Island during the 2012–2013 hunting seasons (Table 1). Since harvests occurred over many congruent days in several locations, we accepted the help of hunters and managers to collect samples and data from their properties. We provided each collector detailed instructions on how to collect tissue and data.

**Table 1:** Wild and captive sika deer sample counts and types from the Delmarva Peninsula, USA, and Yakushima, Japan

Region	Location	Samples	Count & type	mtDNA species confirmation	Microsatellite analysis
Japan	Japan	Yakushima	14 Tissue	14	14
Captive	Delaware, USA	Captive	12 Fecal	12	12
Delmarva	Maryland, USA	Dorchester	55 Tissue	54	54
Delmarva	Maryland, USA	Assateague	30 Tissue	29	29

Note

Counts of samples that were included from each group in each type of analysis are also listed.

We collected fecal samples from 12 captive sika deer (6 fecal samples from an all‐male pen and 6 from an all‐female pen) held in Harrington, DE, USA (Table 1). We collected only fecal samples that were fresh (e.g., pellets were wet, shiny, and covered in a thick mucosal layer) or where we witnessed defecation.

We placed all samples (fecal, muscle, and liver) into sterile 50‐mL centrifuge tubes containing 25 ml of 95% ethanol (enough to cover the sample); we changed gloves between samples. For all samples, we recorded the collection date, harvest location, sex of deer, and species of deer on the 50‐ml tube as well as on paper with pencil inside the tube. We stored all samples in a dark cabinet until we conducted DNA extraction. We extracted DNA from samples with commercial (Qiagen or Bioline) extraction kits for the appropriate sample type and according to the manufacturer's instructions for maximum quantity yield.

We obtained 14 DNA samples from the Yakushima Island sika deer population, Japan, the hypothesized source of the free‐ranging sika deer on the Delmarva (Table 1), courtesy of Dr. H. Tamate and associates from Yamagata University. Samples were shipped as desalted pellets and were resuspended and diluted to a concentration of 100 ng/µl. We stored all DNA at −80°C until analysis.

### 2.1 Identification of species

On Assateague Island and in Dorchester County, sika deer and white‐tailed deer are sympatric and hunting seasons are concurrent. To confirm that the samples collected were sika deer, we performed a restriction fragment length polymorphism (RFLP) assay for each sample (*n* = 109).

We identified a set of primers that amplifies a section of the mitochondrial D‐loop, resulting in a fragment of ~464 base pairs (bp) in length for both sika deer (Wolf, Rentsch, & Hübner, 1999) and white‐tailed deer. We identified differences in base‐pair composition of the sequences using the National Center for Biotechnology Information (NCBI: GenBank) for both Yakushima sika deer and white‐tailed deer. We slightly modified the light strand primer from Wolf et al. (1999), L14735, at two separate base‐pair point changes: transitions at position 9 C to T, and position 16 T to C. Our modifications to Wolf et al. (1999) produced a better match to both Yakushima sika deer and white‐tailed deer genomes (sika deer: Wada, Nishibori, & Yokohama, 2007; white‐tailed deer: Seabury et al., 2011).

5′: aaa aac ca t cgt tgt c at tca act a.

The heavy strand primer (H15149) differed in two positions for sika deer and one position for white‐tailed deer (Seabury et al., 2011; Wada et al., 2007), all at unique locations, and therefore, we did not modify it. We amplified this fragment using the polymerase chain reaction (PCR), with an initial 3‐min denaturation at 96°C, followed by 45 cycles (96°C for 30 s, 60°C for 1 min) and final extension at 72°C for 3 hr (Wolf et al., 1999). The reaction mix included 5 µg of each primer (Invitrogen), 0.05 µg bovine serum albumin (BSA, Promega), 12.5 µl MyTaq Red Mix (Bioline), about 50 ng DNA template, and enough deionized (DI) water to bring the final volume up to 25 µl.

Based on the sequence comparisons (Seabury et al., 2011; Wada et al., 2007), we selected restriction enzyme HinF1, which cut fragments that were large enough to be viewed easily on an agarose gel. HinF1 cut G|AN(R = [A])TC in white‐tailed deer, resulting in two fragments (198 and 265 bp), but did not cut the sika deer fragment (Figure S1). Our restriction cocktail consisted of 5 units of HinF1, 0.025 µg BSA (Promega), 2 µl RE 10X Buffer (Promega: included with enzyme), and 5 µl of the PCR products, and was brought to a final volume of 10 µl with DI water. We digested the samples at 37°C for 3 hr and then held them at 4°C. We electrophoresed the products on a 2% agarose gel containing ethidium bromide (5 µl per 100 µl of agarose mix) and visualized results under UV light.

### 2.2 Genetic diversity and population structure

We evaluated 16 microsatellite DNA loci developed for cattle (*Bos taurus*), sheep (*Ovis aries*), or reindeer (*Rangifer tarandus*) that successfully amplified in multiple species of cervids (Anderson et al., 2002; Bishop, Kappes, Keele, & Stone, 1994; McDevitt et al., 2009; Okada & Tamate, 2000; Tamate et al., 2000; Wilson, Strobeck, Wu, & Coffin, 1997) and displayed high polymorphism and low genotyping error rates (Table S1). Due to low‐quality results in preliminary analysis, we omitted six of the loci and used the remaining 10 for all samples. We carried out amplifications as follows: 4 min denature at 96°C followed by 35 cycles of (30 s denature at 96°C, 60 s annealing (specific temperatures listed in Table S1), and 90 s extension at 72°C) and a final extension of 1 hr at 72°C before being held at 4°C. We created our PCR reaction mix using 10 µl PCR Master Mix (Promega: 400 µM each dNTP; 3 mM MgCl_2_), 5 µM of both forward and reverse primers, 0.05 µg BSA (Promega), 50 ng template DNA, and enough DI water to bring the final volume to 20 µl per sample. We amplified all loci individually and combined them post‐PCR for fragment analysis. We used primers fluorescently labeled at the 5′ end with Applied BioSystems G5 filter set (see Table S1 for dyes) and ran the samples on ABI 3730 DNA analyzer at the Delaware Biotechnical Institute. We scored alleles using GeneMapper Software 3.7 (Applied BioSystems). We only scored peaks in GeneMapper that were 100 relative fluorescence units (RFU's) or greater. Low allele peaks (<300 RFU's) were not common and we only accepted them when they were alleles that were already common in the population (following a relative threshold of calling: Whitlock, Hipperson, Mannarelli, Butlin, & Burke, 2008). We scored heterozygous alleles when the second peak was within 50% of the primary peak's RFU size. All RFU allele‐like peaks that fell within the expected range for the loci but were less than 3X the background noise were scored as missing.

Null alleles may bias downstream genetic analyses (Dąbrowski et al., 2014), and heterologous markers have a greater potential for null alleles. Therefore, we estimated null allele frequencies and mean error rates via Dempster's EM method (Dempster, Laird, & Rubin, 1977) with the program GENEPOP (Raymond & Rousset, 1995; Rousset, 2008).

We indexed genetic variation among populations based on allelic richness and observed heterozygosity. We compared the observed levels of heterozygosity to expected values by testing for Hardy–Weinberg equilibrium using the program GENEPOP (Raymond & Rousset, 1995; Rousset, 2008). For all loci that had four alleles or fewer per population, we used the Fisher's exact test. For locus OarFCB193, we used a Markov chain method at 100 batches and 1,000 iterations per batch. We determined significance for both tests at the 0.05 level (Goodman et al., 2001). The number of observed alleles in a population is influenced by sample size. Since our sample sizes from each area were different, we quantified genetic diversity and estimated private alleles for each population using a rarefaction procedure in the program HP‐Rare based on our smallest sample size (Kalinowski, 2004, 2005).

We used an analysis of molecular variation (AMOVA) based on Wright's F‐statistics (*F*_ST_, *F*_IT_, and *F*_IS_) implemented in the program GenAlEx (Peakall & Smouse, 2006, 2012; Wright, 1965). Measurements of F‐statistics were calculated across all 10 loci, and statistical significance was assessed based on 999 permutations among individuals or populations, as appropriate. We also estimated genetic diversity within individuals, within populations, between populations, and between geographical regions with GeneAlEx.

We estimated population structure among sika deer from Delmarva, Japan, and the captive deer using multivariate and Bayesian clustering analyses. We performed a principle coordinate analysis (PCoA) to compare genetic similarity of individuals within and among populations. We performed the PCoA on matrices of genetic distances between individuals based on a converted covariance matrix using the computer program GeneAlEx (Peakall & Smouse, 2006, 2012). We also estimated population structure with a Bayesian clustering analysis performed in the computer program STRUCTURE (Prichard, Stephens, & Donnelly, 2000). We used a burn‐in of 100,000 Markov Chain Monte Carlo reps followed by 1,000,000 iterations of data collection. We estimated clusters (K) from 1 through 7, with 8 repetitions of each K. We determined the best‐fit cluster solution using a modification of the Evanno method (Evanno, Regnaut, & Goudet, 2005) implemented in the computer program STRUCTURE HARVESTER (Earl & vonHoldt, 2012).

### 2.3 Demographic history

We used approximate Bayesian computation (ABC) to make inferences about wild sika deer on the Delmarva including the potential for serial founder events, using the computer program DIYABC v 2.0.4 (Cornuet et al., 2008). Approximate Bayesian computation analysis can model complex population histories, including bottlenecks or founder events, changes in population sizes, and admixture. We quantified support for demographic scenarios by generating simulated posterior probability models based on given demographic priors in a coalescent framework (Cornuet et al., 2008; Lawson Handley et al., 2011). We selected uniform probabilities for all scenarios on all parameters.

All priors provided in DIYABC scenarios were based on the best information available regarding historic and current status of wild sika deer on the Delmarva and Yakushima Island, Japan, including a proportional buffer (Table 2). We estimated current effective population sizes based on total population estimates for Dorchester, Assateague, and Yakushima, Japan; we assumed that Yakushima, Japan samples represent the allelic diversity of the founding stocks (Table 2). The DIYABC output includes estimates of all parameters for timing (in generations: T*_i_*), duration of bottlenecks (in generations: db*_i_*) and population sizes after bottlenecks or founder event (Nf*_i_*).

**Table 2:** Parameters used as priors for approximate Bayesian computation with known information for sika deer introduced to the Delmarva Peninsula, USA

Event	Parameter	Prior information used for approximate Bayesian computation analysis			Resulting posterior estimates from ABC			
		Information	Prior	Citation	Mean	Median	q050	q950
Ne Dorchester	Dorchester	Annual harvest is 2,500 for Dorchester alone. Population estimate for Maryland is ~ 10,000–12,000 with the bulk of individuals in the Dorchester population.	250–2,500	Eyler and Tymko (2014)	1,300.0	1,230.0	414.0	2,340.0
Ne Assateague	Assateague	See above	80–1,125	USFWS (2014)	156.0	111.0	83.5	403.0
Ne Yakushima, Japan	Japan	Population between 2,000 and 18,000 (Ne:200–4,500)	200–5,000	Tsujino, Noma, and Yumoto, (2004)	3,070.0	3,200.0	952.0	4,840.0
Ne Ghost	Ns	Number of deer that contributed to admixture while in UK.	2–20 ind.	No account provided	11.0	11.1	3.0	19.0
Fire ‘57	t1	1957 (59 ybp)	10–30 gen.	Flyger and Bowers, (1958)	13.9	12.7	10.0	21.8
Fire Lag length	Db	population remained small for several generations	2–7 gen.	Presnall (1958)	5.7	6.3	2.0	7.0
Fire Ne	Nf1	160 total deer (Ne: 16–40)	14–45	Flyger and Bowers, (1958)	28.3	27.7	15.1	43.1
Assateague split	t2	Assateague founded. 1924 (91 ybp)	15–45 gen.	Flyger (1960)	21.7	20.3	15.4	33.4
MD founding	t3	Arrival/ release into wild. 1916 (99 ybp)	16–50 gen.	Flyger (1960)	35.8	36.0	22.7	47.7
MD lag	db2	Uncertainty about time between actual arrival and release into wild. Population remained small for several generations	2–10 gen.	Presnall (1958)	6.1	6.1	2.0	10.0
MD founders	Nf2	4 or 5 individuals * (such a small founding population we allow for the entire count)	1–5 ind.	Flyger, (1960)	3.6	4.3	1.0	5.0
UK Woburn Abbey	t4	Arrival in England 1893 (123 ybp)	20–61 gen.	Bedford, (1949), Lowe and Gardiner (1975)	45.3	45.9	29.4	58.7
Admixture	r1	Some degree of admixture, degree relatively unknown	0.001%–0.3%	Bedford, (1949), Banwell, (1999)	0.138	0.132	0.013	0.282
Ireland Founding	t5	Arrival in UK (Ireland) 1,860 (155 ybp)	26–78 gen.	Powerscourt (1884)	74.6	75.6	68.0	78.0
UK lag	db3	Time spent in England. Estimated from establishment in Woburn Abbey through arrival in US. 23 years	4–11 gen.	Lowe and Gardiner (1975)	2.1	1.8	1.0	5.0
UK founders	Nf3	Number of deer that contributed to Founding of UK from Yakushima Island deer	4 ind.	Powerscourt (1884)	3.4	3.9	1.0	4.0
Yakushima Founded	t6	Time spent on Yakushima Island after glaciation split, expected to fall between t5 and t7 generations.	78–10,000	No account provided	4,700.0	4,520.0	480.0	9,370.0
Yakushima Founders	Nf4	Population size at split during glaciation.	20–5,000	No account provided	24.1	23.2	3.8	47.2
Sika split	t7	Time when "ghost" population and Yakushima population are expected to have diverged (Riss‐Würm interglacial period: 100,000 ybp)	±25% error. 12,500–62,500 gen.	Tamate et al. (1998)	33,500.0	31,700.0	14,100.0	58,600.0
Mutation rate	Âµmic_1				9.04E‐04	9.27E‐04	7.30E‐04	1.00E‐03

Note

Effective population sizes were estimated as between 10% and 25% (Palstra & Fraser, 2012) of total population estimates when provided unless otherwise noted. Resulting posterior estimates shown as means, medians and 95% and 5% bounds.

For each ABC analysis we created one million simulated data sets per scenario, using the same number of loci and individuals as the original data set. We ran all mutation rate (µ) models with prior distributions for a generalized stepwise mutation model (GSM) for each locus (uniform mean mutation rate: 1.00E‐004 min, 1.00E‐3 max; uniform coefficient *p*: 1.00E‐001 min, 3.00E‐001 max; gamma individual mutation rate: 1.00E‐005 min, 1.00E‐002 max; and gamma individual coefficient *p*: 1.00E‐002 min, 9.00E‐001 max). We calculated the relative confidence in each set of scenarios via polychotomous logistic regression using the best 0.1 proportion of the data sets simulated. We calculated posterior distributions from this top 0.1 proportion of the data from the best scenario using linear regression of the logit‐transformed results (Cornuet et al., 2008).

We considered a range of demographic scenarios in varying complexity, from simple independent introductions without admixture and a single founder event (three historic events), to introductions from admixed populations with multiple founder and bottleneck events (nine historic events and 19 separate parameters). All of our demographic models included a split between the two wild portions of Delmarva sika deer, Dorchester and Assateague (timing and length of founder event changed). All demographic models involved a bottleneck in the Dorchester population, and all demographic models involved these two populations splitting after the stock was founded from the Yakushima Japanese population. We compared scenarios in groups of 2–4, changing single parameter events (e.g., did bottleneck happen at the same time period as the introduction or were there two separate events) and compared the best scenario from each for a final best case estimation of timing and magnitude of demographic events.

Our simplest scenario was wild Delmarva sika deer were founded directly from the Yakushima, Japan population: a population of sika deer that split into modern Yakushima, Japan samples and a second branch that initiated wild Delmarva sika deer and later split into isolated Dorchester and Assateague sika deer. The bottleneck in the Dorchester population happens after the split from Assateague deer in this scenario. Our second scenario was that wild Delmarva sika deer are from a different stock of sika deer, but incorporated some genetic admixture with sika deer from Yakushima, Japan; a population of sika deer that splits forming an “unknown” population, and the Yakushima, Japan population. Individuals from these populations meet and their offspring are a combination of the genetic lineages of both. The third scenario involves the Yakushima, Japan sika deer as the parent lineage to wild Delmarva sika deer, but there was introgression of genes from another stock in the recent past; the Yakushima, Japan sika population split from an unknown population, followed by a series of founder events. Individuals from a branch of the Yakushima Island line meet some from the unknown line and this new lineage forms the base of the branch for the introduced wild Delmarva sika deer.

To verify the performance of the selected model, we used the model check option in DIYABC to estimate the goodness of fit of the simulated data sets with our original data (Cornuet et al., 2014). Model check was based on 18 summary statistics across all three original populations including mean number of alleles, allele size, *F*_ST_, mean genetic diversity, shared allele distance, and (δµ)^2^, a measure of genetic distance between populations. We also estimated our confidence in the selected model using linear discriminant analysis of the summary statistics to provide a confidence interval to differentiate between sets of scenarios (Cornuet et al., 2014).

## 3 RESULTS

We know that each sample was from a unique individual because all wild Delmarva samples were collected from harvested sika deer, there are roughly 12,000 free roaming throughout the Peninsula (Kalb & Bowman, 2017). Based on microsatellite results, we were able to identify each of the captive sika deer as a unique individual. All of the sika deer samples from Yakushima, Japan, were collected from unique individuals.

All but two of our samples were identified as sika through RFLP analysis; one from each Dorchester and Assateague identified as white‐tailed deer or did not amplify. We genotyped 109 total samples; 12 captive sika deer, 14 Yakushima, Japan, 29 wild sika deer from Assateague Island, and 54 wild sika deer from Dorchester County. We observed 41 alleles across the 10 loci, with more alleles in both the Yakushima, Japan, and captive sika deer samples than in either of the wild Delmarva populations (Assateague and Dorchester); we used rarefaction to compensate for small sample sizes and compare allelic richness (Table 3). We randomly selected and repeated samples to regenotype and scored them separately (blind error rate: Bonin et al., 2004). The error rate for scored genotypes was 4.6% based on 283 repeated loci out of 1,409 scored loci. Our estimated error rate (program GENEPOP) was 4.9% based on population‐locus combinations with more than 3 alleles per locus. Our genotypic data set was 93% complete across all 10 loci, with 48% of missing genotypes observed at locus BM1225.

**Table 3:** Estimated allelic richness and private alleles from rarefaction analysis in the computer program HP‐Rare in samples from wild Delmarva Peninsula, USA sika deer (Assateague and Dorchester), captive sika deer (Delaware, USA) and from the source population of the wild sika stocks (Yakushima, Japan)

	Assateague (29)	Dorchester (54)	Japan (14)	Captive (12)
Allelic richness	1.1	1.22	1.96	2.28
Private alleles	0	0.03	0.54	0.99
Allele count	11	14	25	28

Note

Total alleles observed in each population (*N* samples) from this study are included.

Allelic diversity ranged from one allele (OarFCB304) to eight alleles (OarFCB193) between populations, with a mean of four alleles per population (Table S1). Most of the observed allelic diversity was within captive sika deer and Yakushima Japanese samples (29 of 41 observed alleles). We observed two private alleles, one at each marker, IGF‐1, and BM203 in the wild Delmarva sika deer, both of these were found in the Dorchester samples. No private alleles were observed in Assateague samples. The Yakushima Japan samples had 11 private alleles and captive sika deer samples had 10 private alleles (Table 3).

Expected and observed heterozygosity deviated from Hardy–Weinberg equilibrium expectations in five population‐locus combinations (Table 4). Both BM1225 and OarFCB193 displayed a deficit of heterozygotes in the Yakushima Japanese samples, RT27 was heterozygote‐deficient in the captive sika deer samples, and IGF‐1 was heterozygote‐deficient in the wild Dorchester samples. One locus, OBCAM, displayed an excess of heterozygotes in the wild Dorchester population (Table 4). Mean null allele frequency was 0.13 across 9 loci per population.

**Table 4:** Observed (*H*_O_) and/ expected (*H*_e_) heterozygosity rates across all loci in populations of sika deer from the wild on the Delmarva Peninsula (Assateague and Dorchester), USA, captive sika deer from Delaware, USA, and the source population of the wild sika stocks (Yakushima, Japan)

	Assateague (29)	Dorchester (54)	Japan (14)	Captive (12)
IGF‐1	0.00/0.00	0.00*/0.02	0.04/0.04	0.00/0.11
BM4107	0.00/0.00	0.00/0.00	0.35/0.29	0.23/0.27
OarFCB304	0.00/0.00	0.00/0.00	0.00/0.00	0.00/0.00
BM203	0.13/0.12	0.30/0.25	0.19/0.17	0.05/0.05
BM1225	0.00/0.00	0.00/0.00	0.00*/0.31	0.00/0.33
OBCAM	0.00/0.00	0.32!/0.22	0.20/0.20	0.07/0.06
RT27	0.00/0.00	0.00/0.00	0.04/0.04	0.00*/0.08
BM6438	0.00/0.00	0.00/0.00	0.15/0.14	0.10/0.26
OarFCB193	0.00/0.00	0.00/0.00	0.04*/0.15	0.30/0.37
GM4006	0.00/0.00	0.00/0.00	0.04/0.04	0.14/0.25

Note

Locus and population heterozygosity values not in Hardy–Weinberg equilibrium (HWE): values with * have heterozygosity deficiency, those with ! have heterozygosity excess. Departures from HWE may be a result of missing alleles during the scoring process, which were not evenly distributed between samples or loci.

### 3.1 Population structure

**Figure 3:**
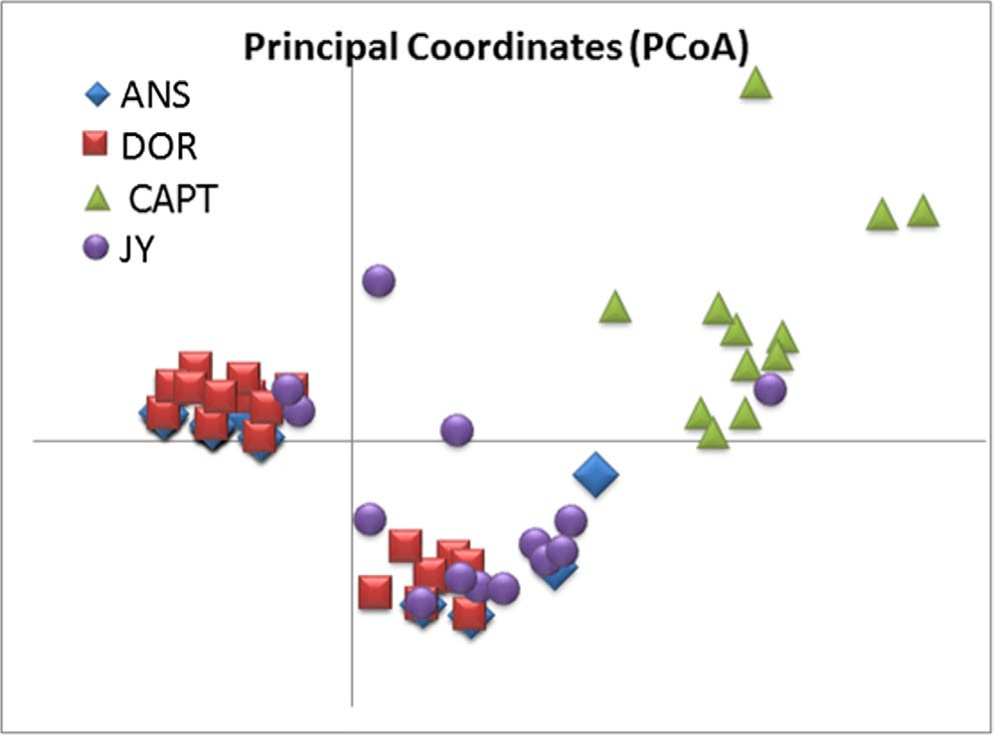
Principle coordinate analysis of percentage of genetic variation across sampling areas of sika deer from the wild on the Delmarva Peninsula (Assateague [ANS] and Dorchester[DOR]), Delmarva Peninsula captive sika deer (captive[CAPT]) and the source population of the wild sika stocks (Yakushima, Japan[JY]). Individual assignment to populations was done using nine polymorphic microsatellites. Across all samples, 44% of the variation in genetic distance was explained with the first coordinate axis, the second axis explained an additional 10% of the variation in our samples, and the third (not shown) an additional 8%

**Figure 4:**
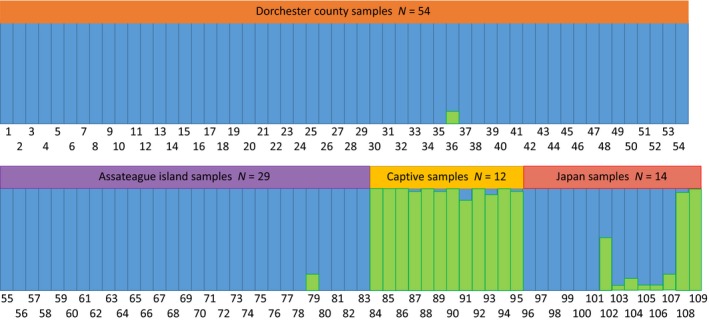
Analysis of Bayesian clustering identified two cluster groups (blue and green) based on 10 loci across all sample populations of sika deer from the wild on the Delmarva Peninsula (Assateague and Dorchester), Delmarva Peninsula captive sika deer (Captive) and the source population of the wild sika stocks (Yakushima, Japan). Population cluster assignment was estimated according to a slight modification of the Evanno et al. (2005) method in STRUCTURE Harvester (Earl & vonHoldt, 2012). Colored bars above each individual are the sampling location, and the numbers are individual deer samples. Orange bar is Dorchester samples *N* = 54, violet bar is Assateague samples *N* = 29, yellow bar is Captive samples *N* = 12, and red bar is Yakushima Japan samples *N* = 14

The AMOVA showed all populations were differentiated from each other based on *F*_ST_, *F*_IS,_ and *F*_IT_ (Tables S2 and S3). Most of the genetic variation of these populations was measured (by *F*_ST_) among populations (54%) or within individuals (45%). In our PCoA analysis, the top two axes explained 51% of the cumulative variation between populations (Figure 3). Wild sika deer (Assateague and Dorchester) samples produced similar, intersecting plots. Sika deer from Yakushima, Japan, both overlapped slightly with wild sika deer samples but were more broadly dispersed across the two axes and only slightly overlapping each other (Figure 3). Samples from captive sika deer clustered together away from the other samples with very little overlap between other populations, but a high degree of overlap among the populations (Figure 3). Bayesian clustering analysis supported two clusters. Captive sika deer samples were clearly differentiated, while the wild sika deer (Dorchester and Assateague) populations formed a separate cluster; the Yakushima Japan samples fit into both clusters (Figure S2, Figure 4). While STRUCTURE HARVESTER can only evaluate two or more populations, given the way this delineated the samples and what is in the literature regarding sika clades from Mainland Asia versus Japanese Islands, we feel the program provided valid clusters (as opposed to all sika deer samples being from a single population).

### 3.2 Demographic history

**Figure 5:**
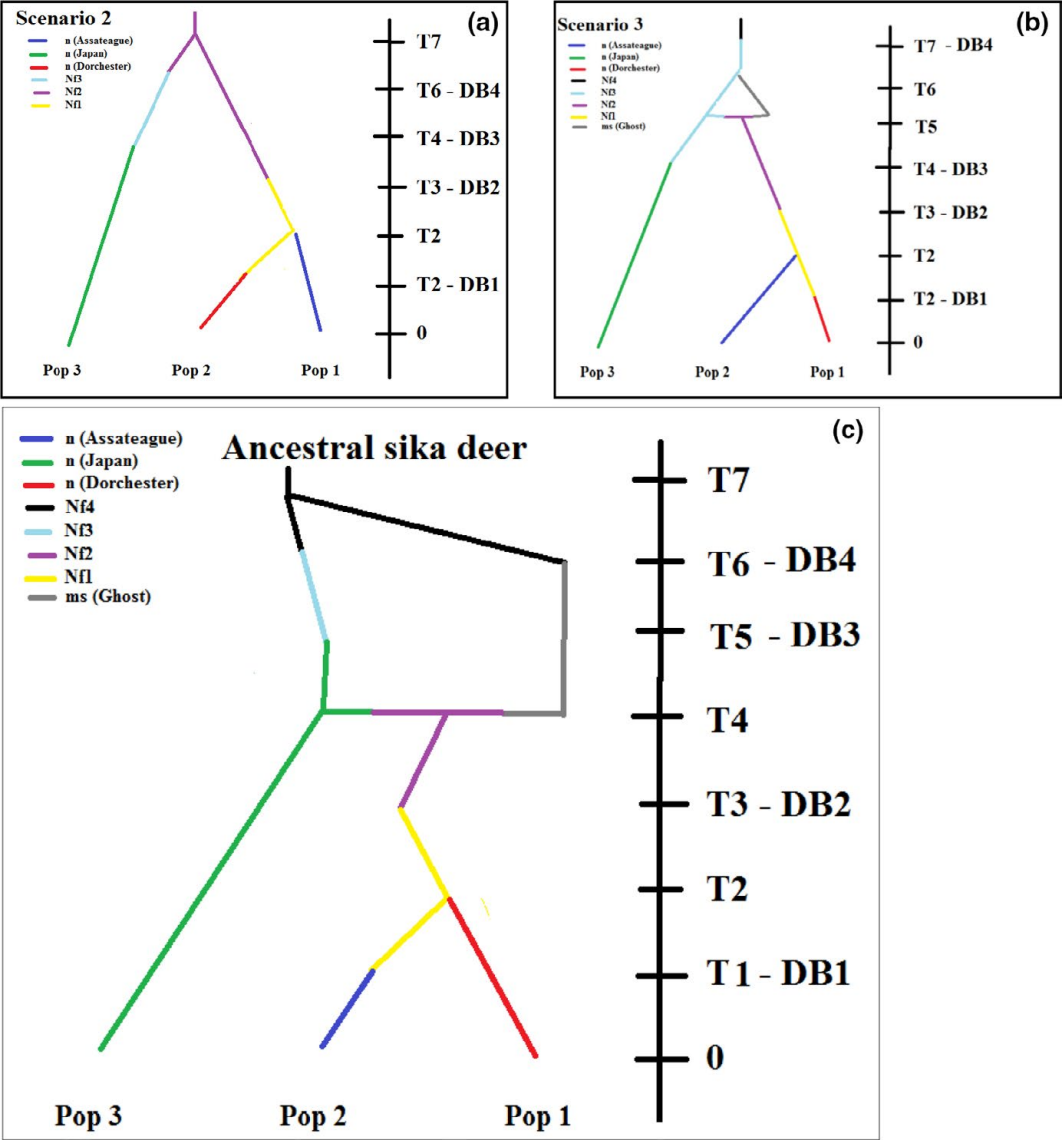
Top historical models selected by DIYABC. Timelines on the right are not to scale. Change in colors within images represents a population split, or a population bottleneck. Time 0 represents the current (collection) date. Image c, (enlarged) was selected as the best of all models. The right side from time periods t3–t6 are equivalent to sika deer being in the United Kingdom, and time periods 0–t3 represent sika deer on the Delmarva Peninsula (wild deer only). The black color represents some ancestral sika deer lineage (labeled ancestral) that through time evolved into two separate lineages with some level of genetic differentiation, the gray color is an unknown population that was not sampled. The transition on the left wing from black to blue and again from blue to green represents a bottleneck or population reduction in the genetic diversity. Where green Japanese lineages meet the gray unknown lineage, there is genetic transfer creating the purple lineage of sika deer. The yellow lineage represents the midlineage population of sika deer that stocked the Delmarva Peninsula with a bottleneck at transition to the blue and red colors. Color schemes are similar within images a and b, but timings differ and represent different potential introduction scenarios. Image a, was a top model from possibilities that did not have any genetic introgression from other sika deer sources (presuming that there was a straight line introduction from either the Japanese Islands or United Kingdom. Image b, was the top model for only ancestoral introgression prior to sika deer populations leaving the Japanese Islands (it includes a gray “ghost” population)

We had three top model scenarios in our approximate Bayesian computation (Figure 5). Comparing and pre‐evaluation of scenarios selected our third model 86% of direct approach estimates and 97% of logistic approach estimates. The most plausible scenario involved seven time stages. Our top model included a ghost population with genetic admixture between time periods 4 and 6 (when sika deer were in the United Kingdom [England and Ireland]). Estimates of posterior population statistics included known values from historic literature (Table 2). Bottleneck durations were longer in the US, 5.7 generations (db) and 6.1 generations (db2), compared with the duration in the UK 2.1 generations (db3).

We estimated that (in reverse order from current date) 28.3 (Nf1) effective breeders survived the fire on James Island which occurred 13.9 (t1) generations ago. The actual year of the fire on James Island was 1957, or 58 years before present, a mean generation time of 4.2 years. The split of the wild Delmarva founding population, from Dorchester to Assateague, was estimated at 21.7 (t2) generations ago. The actual year of the split between Dorchester and Assateague was 1924, a mean generation time of 4.2 years per generation. Our estimate for founder individuals was 3.6 (Nf2), which was estimated at 35.8 generations ago (t3). The introduction of wild sika deer reportedly occurred in 1916, about 2.8 years per generation. Sika deer arrived in England about 45.3 (t4) generations ago from two populations, the ghost population of 11 effective breeders (NS) and Japan deer (Nf3), with 3.4 effective breeding individuals. The sika deer that arrived in England went through a bottleneck on their way from Ireland, an estimated at 74.6 generations ago (t5), in 1884. The sika deer populations that were in England and Ireland at this time derived from several subspecies and mixed stocks; we estimated a coalescence date of 4,700 (t6) generations ago. In our model checking, only 3 of the 18 statistical parameters had simulated data that were less than the observed values, 2 mean number of alleles (in Assateague and Yakushima Japan simulated populations), and 1 mean size variance. The goodness of fit confidence in our selected scenario was high: of 500 simulated scenario estimates, 7 supported scenario A, 3 supported scenario B, and the remainder, 98%, supported our top model scenario C (Figure 5).

## 4 DISCUSSION

The amount of genetic diversity maintained through founder events is influenced by the number of breeding individuals, drift and other chance events, and the rate of population growth postintroduction (Estoup, Wilson, Sullivan, Cornuet, & Moritz, 2001). Wild sika deer on the Delmarva were founded by few individuals, from source stocks with limited diversity. The introduced population experienced slow growth and postintroduction bottlenecks, resulting in populations with further reduced neutral genetic diversity.

Sika deer on many Japanese Islands have lower genetic variation than observed in mainland Asian populations of cervids (Lü, Wei, Li, Yang, & Liu, 2006). The colonization of Japan by sika deer through the rise and fall of sea levels during the Riss‐Würm (North American equivalent: Sangamon) interglacial and Würm (North American equivalent: Wisconsinan) glacial periods of the Pleistocene produced small, isolated populations of sika deer (Tamate & Tsuchiya, 1995). These small populations display limited variation within most populations, as well as genetic differentiation between isolated populations (Goodman et al., 2001; Nagata, Masuda, Kaneko, & Yoshida, 1998; Nagata et al., 1999; Tamate, 2009). These geographic separations translated to captive populations on the Delmarva that are both physically (substantially taller and heavier) and genetically very different from the wild populations implying that the captive animals were sourced from several different provinces (Kalb & Bowman, 2017).

We observed more alleles and a greater number of private alleles in the captive sika deer samples compared to both the Yakushima, Japan samples and wild Delmarva sika deer samples (Table 3). The high proportion of private alleles found between Yakushima, Japan, and captive sika deer samples is likely a result of the historic separation between mainland Asian sika deer and Japanese sika deer, and because the captive sika deer are a mixture of different sika deer stocks some of which were known to be from Manchurian (mainland) decent (Olson, Whittaker, & Rhodes, 2013).

We observed a decline in genetic variation in populations congruently with the timing and pattern of establishment. In an increasing order of genetic variation, wild sika deer on Assateague were founded from wild deer in Dorchester, founded from stocks in England and Ireland, which were derived from Japanese sika deer (Goodman et al., 2001; Nagata, Masuda, Kaji, et al., 1998; Senn, 2009). While different loci were used in their evaluation, Senn (2009) and Nagata, Masuda, Kaji, et al. (1998) also observed a pattern of low genetic variation suggesting a long‐term, serial loss of diversity in sika deer through bottleneck and founder events.

While most of the alleles that we observed in wild Delmarva sika deer populations were found in the Yakushima Japan samples (12 of 14), there were two private alleles. These data support three hypotheses about the wild sika deer on the Delmarva. The first is that the primary contributing population is the Yakushima Island subspecies (*C. n. yakushimae*). The second is that there may have been genetic admixture with other stocks prior to the introduction onto the Delmarva. Antecedent sika deer to the wild Delmarva from Ireland and England had the chance to interbreed with multiple other deer species and subspecies (Bedford, 1949). Alternatively, the alleles may exist in Yakushima but were not present in this sample. The private alleles may have been lost from Yakushima but retained in Delmarva, which seems unlikely because more variation should be lost from the Delmarva deer during the multiple founding events and bottlenecks. It is also possible that the alleles are novel and derived from mutation after introduction. Finally, the low allelic diversity and proportion of shared alleles observed in both wild Delmarva sika deer populations and the source stock in Yakushima, Japan, is consistent with a single introduction and subsequent founder event.

Genetic variation we observed in Delmarva sika deer is similar to other populations of ungulates that have been founded from few individuals. For instance, introduced populations of elk in Pennsylvania and white‐tailed deer in Finland display reduced variation relative to the source stocks (Kekkonen, Wikström, & Brommer, 2012; Williams, Serfass, Cogan, & Rhodes, 2002). Elk in Pennsylvania showed 7 of 10 loci were fixed or had been reduced to two alleles (Williams et al., 2002). White‐tailed deer introduced to Finland maintained greater allelic richness and higher heterozygosity (5.36, 0.692) across 14 loci than observed in sika deer and elk; however, they were founded from deer that were more highly variable (Kekkonen et al., 2012). The population in Finland also received additional genetic variation from a secondary introduction 14 years postfounding and continued to grow, providing additional chance for genetic drift (Brommer, Kekkonen, & Wikström, 2015; Kekkonen et al., 2012).

Similar to the translocation of bighorn sheep (*Ovis canadensis*), subsequent establishments of sika deer in the wild populations of the Delmarva show progressive decline in neutral genetic variation (Olson et al., 2013). Wild sika deer of the Delmarva were founded from a single introduction of stocks with low diversity, followed by a lag in population growth and at least one bottleneck event. Additionally, since the population of wild sika deer on the Delmarva remained small for several generations, there was a potential for the loss of neutral genetic variation due to genetic drift.

Reductions in genetic diversity due to inbreeding can lead to reduced sperm count, decreases in birth rates, decreases in juvenile survival, and increased susceptibility to disease (Lawson Handley et al., 2011; O'Brien et al., 1985; Sakai et al., 2001). However, wild Delmarva sika deer have proliferated and in some cases replaced native white‐tailed deer. Wild sika deer were observed to have lower susceptibility to parasites (tics and other insects) and disease than native white‐tailed deer (Davidson & Crow, 1983) in spite of their lack of genetic diversity. Sika deer on the Delmarva have also been successful reproducing, as emphasized in their near exponential population growth early in the introduction, and extensive, and increasing annual harvest throughout their expanding range (Kalb & Bowman, 2017).

Therefore, despite low neutral diversity, sika populations appear to be robust. In some cases, adaptive diversity can be maintained if the forces of selection outpace genetic drift, or if highly favorable genes are fixed via “allele surfing” (Hedrick, 2004; White, Perkins, Heckel, & Searle, 2013). It is possible that introduced sika have retained sufficient adaptive variation to be successful in their new environment.

Limited genetic diversity precludes fine‐scale inferences on population substructure and assignment on the Delmarva sika deer. We calculated high values (Balloux & Lugon‐Moulin, 2002; Wright, 1978) for *F*_ST_ and *F*_IT_ as a result of the complex nature of the introduced wild sika deer to the Delmarva. The calculated *F*_IS_ values were not as high (Table S3), but do suggest some degree of population structure within the sampling area due to social behavior or likely geography; such values may also be a result of allele amplification issues. The assignment into population clusters also confirms a lack of gene flow between captive sika deer and the wild sika deer on the Delmarva. The Yakushima Japanese samples were found to have mixed population assignment with some individuals more similar to captive sika deer samples and some individuals more similar to the wild sika deer samples.

While approximate Bayesian computation is sensitive to low sample sizes, the results strongly supported the recorded history of the introduction of wild sika deer to the Delmarva. The best‐fit demographic scenario for our computational analysis involves a single introduction of sika deer forming the wild population. The rate of genetic admixture in our best‐fit scenario suggests that there was little introgression of new genes in sika deer that were in the United Kingdom. The duration of bottleneck events were much longer in the US (Table 2: db and db2 vs. db3) than in England or in Ireland. This further supports how wild sika deer on the Delmarva generally went unnoticed in the first decades postintroduction. On the contrary, sika deer introduced to Ireland expanded quickly (Powerscourt, 1884). In the US, the range and population size of sika deer were most likely restricted from an invasive lag. We define invasive lag as a specific time frame between introduction and establishment of the invader. This could be years or decades before the population begins to experience rapid growth and range expansion consistent with invasive species. In the case of sika deer on the Delmarva, it appears that the lag period was a few generations and was probably increased in length due to the fire event and population collapse.

Generation estimates from ABC follow a similar growth rate estimated in bottleneck durations. The average years per generation were also less in the UK (2.7) than were estimated in the US (4.2) based on known dates (Table 2: t1 and t2 vs. t3 and t4) but were consistent between regions. These generational times fit within the known ecology of the species. Longer periods of time, with small population size, increase the effects of random drift on the population and could have resulted in the loss of some neutral genetic variation. Until recently, the expansion of wild sika deer northward up the Delmarva has been fairly limited. We feel our results support the continued application of ABC for lineage investigations when sufficient evidence is available to create the proper parameters.

From an original introduction of approximately five individuals, the population of wild sika deer of the Delmarva has increased to an estimated 10,000–12,000 and has spread through seven counties, as well as into neighboring Delaware and Virginia. The spread of sika deer across Delmarva continues with new sightings and reports of harvests outside their range in Maryland and Delaware nearly every year despite the desire to see their spread halted. Due to the close evolutionary history between sika deer from Assateague and Dorchester areas, the current microsatellite suite will not be informative in determining how sika deer expand from either core source. The Maryland Department of Natural Resources has tried to manage sika deer as a local economic and social benefit to the communities of Dorchester and Worchester. Recently, harvest bag limits were increased for the 2014–2015 hunting season, and harvest management will be the primary means of controlling sika deer.

The continued growth and expansion of sika deer range, and increasing population size despite reduced genetic variability are an interesting study example of the genetic paradox of invasive species (Estoup et al., 2016; Frankham, 2005). The timing of introduction relative to sika deer exhibiting invasive behavior, however, was likely restricted by several large population bottlenecks and resulting in a major invasive lag. The population at large, throughout the Delmarva provides a semigeographically isolated and intensely managed study and research opportunity and we recommend its continued utilization for large scale, terrestrial invasive research.

We also encourage the Delaware Department of Natural Resources to design and implement management protocols specifically for sika deer, which should include liberal harvest regulations and ample administration of permits for agricultural damage (ideally to prevent sika deer spread through the state). Most importantly, Delaware officials will need to address the future of captive sika deer on the Delmarva, and if the potential risks associated with an escapee or released animal are warranted.

Future studies regarding sika deer genetics would benefit from a review of known restorations and introductions to help identify available markers that may be more informative at the population level. Studies within the Delmarva could focus on using a wider set of microsatellite loci and evaluating single‐nucleotide polymorphisms, which would be informative in addressing if the sika deer of the Delmarva have evolved or maintained any adaptive diversity during their founding. We also suggest a more thorough sampling of sika deer on the Delmarva, especially in Wicomico, MD, and Sussex, DE to evaluate the spread of sika deer as individuals from the two wild populations, Dorchester and Assateague, begin to meet again.

## CONFLICT OF INTEREST

None declared.

## AUTHOR CONTRIBUTIONS

This paper is part of the doctoral research of David Kalb at the university of Delaware department of Entomology and Wildlife Ecology. Dr. Bowman, Dr. Delaney, and Dr. DeYoung all served on the dissertation committee and were contributors to all parts of the manuscripts success, from conception to completion.

## Data Availability

Sampling locations and microsatellite genotypes will be stored in Dryad when manuscript is accepted. Primary author is register with Dryad. Dryad https://doi.org/10.5061/dryad.54m0128
