# Supplementary material for: Genetic diversity and demographic history of introduced sika deer on the Delmarva Peninsula
Source: Ecol Evol. 2019 Sep 27;9(19):11504–17. doi: 10.1002/ece3.5655 (PMC6802040; doi:10.1002/ece3.5655)
Supplement: Supplementary file 1 [file ECE3-9-11504-s001.docx]

**Supplementary Tables and Figures**

Table S1. Microsatellites used to determine population structure and genetic variation of sika deer on the Delmarva Peninsula with total number of alleles observed from all populations, the dye and colors, annealing temperatures for amplification, and grouping of markers for reading.

_______________________________________________________________

| **Locus** |  | **Total Alleles** | **Dye/color** | **Annealing Temp** | | **Group** | |  |
| --- | --- | --- | --- | --- | --- | --- | --- | --- |
| OarFCB193 |  | 8 | NED-Yellow | 58 | 1 | |  | |
| OarFCB304 |  | 1 | VIC-Green | 58 | 2 | |  | |
| RT27 |  | 3 | PET- Red | 55 | 2 | |  | |
| OBCAM |  | 3 | NED-Yellow | 55 | 2 | |  | |
| BM4006 |  | 3 | VIC-Green | 54 | 1 | |  | |
| BM6438 |  | 5 | 6FAM- Blue | 59 | 1 | |  | |
| BM1225 |  | 4 | PET- Red | 56 | 3 | |  | |
| BM4107 |  | 5 | 6FAM- Blue | 54.5 | 3 | |  | |
| BM203 |  | 4 | 6FAM- Blue | 56 | 2 | |  | |
| IGF-1 |  | 4 | NED-Yellow | 56 | 3 | |  | |
| RM188 |  | NA | VIC-Green | 54 | NA | |  | |
| TGLA126 |  | 2 | 6FAM-Blue | 56 | NA | |  | |
| TGLA127 |  | 1 | NED-Yellow | 54 | NA | |  | |
| TGLA337 |  | NA | PET-Red | ? | NA | |  | |
| IDVGA55 |  | 1 | NED-Yellow | 56 | NA | |  | |

Table S2. Degree of genetic differentiation among populations (F_ST_) describing genetic structure of samples of sika deer from the wild on the Delmarva Peninsula (Assateague and Dorchester), Delmarva Peninsula captive sika deer (captive) and the presumed source population of the wild sika stocks (Yakushima, Japan) as measured by GeneAlEx. Values below the diagonal are F_ST_, values above the diagonal are P values from AMOVA from GeneAlEx.

___________________________________________________

|  | **Assateague** | **Dorchester** | **Captive** | **Japan** |
| --- | --- | --- | --- | --- |
| **Assateague** |  | 0.001 | 0.001 | 0.001 |
| **Dorchester** | 0.094 |  | 0.001 | 0.001 |
| **Captive** | 0.624 | 0.633 |  | 0.001 |
| **Japan** | 0.220 | 0.255 | 0.302 |  |
|  |  |  |  |  |

Table S3. F-statistics from all loci with all sika deer populations pooled: Dorchester (N=54), Assateague (N=29), Captive (N=12) and Japan (N=14) pooled. Statistics marked with an ^*^ significant (ɑ = 0.05) from AMOVA performed in the program GeneAlEx.

___________________________________________________________________________________________________

|  | **IGF-1** | **BM4107** | **OarFCB304** | **BM203** | **BM1225** | **OBCAM** | **RT27** | **BM6438** | **OarFCB193** | **GM4006** | **Total** |
| --- | --- | --- | --- | --- | --- | --- | --- | --- | --- | --- | --- |
| **Fst** | 0.025 | 0.674^*^ | N/A (monomorphic) | 0.307^*^ | 0.721^*^ | 0.246^*^ | 0.093^*^ | 0.796^*^ | 0.631^*^ | 0.566^*^ | 0.544^*^ |
| **Fis** | 0.682^*^ | -0.052 | N/A (monomorphic) | -0.098 | 0.254^*^ | -0.366 | 0.653^*^ | 0.109 | 0.329^*^ | 0.387^*^ | 0.016 |
| **Fit** | 0.690^*^ | 0.657^*^ | N/A (monomorphic) | 0.239^*^ | 0.792^*^ | -0.029 | 0.685^*^ | 0.818^*^ | 0.752^*^ | 0.734^*^ | 0.551^*^ |

___________________________________________________________________________________________________


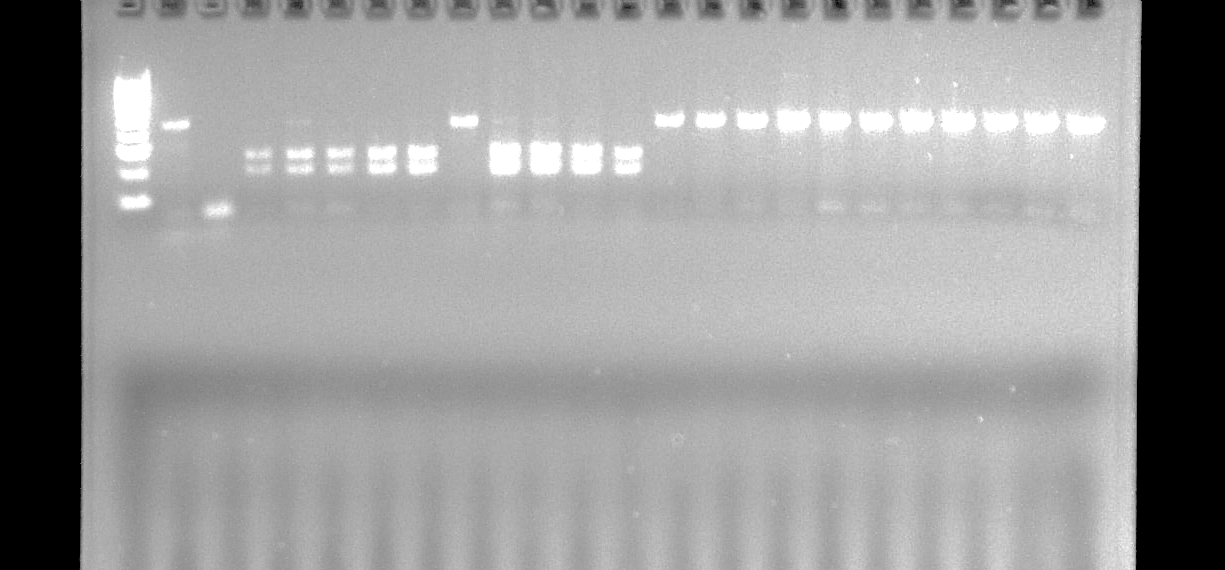


Fig. S1 Restriction fragment length polymorphism restriction results to confirm the species of samples that were collected by hunters in Maryland, where sika and white-tailed deer co-occur. Lane 1: 1000bp ladder, lane 2: + control sika, lane 3: – control PCR cocktail, lanes 4-8, and 10 – 13 are white-tailed deer which was cut by restriction enzyme HinF1 into two pieces (198+265bp), lane 10 and 14 – 24 are sika deer which were not cut by the enzyme (464bp).


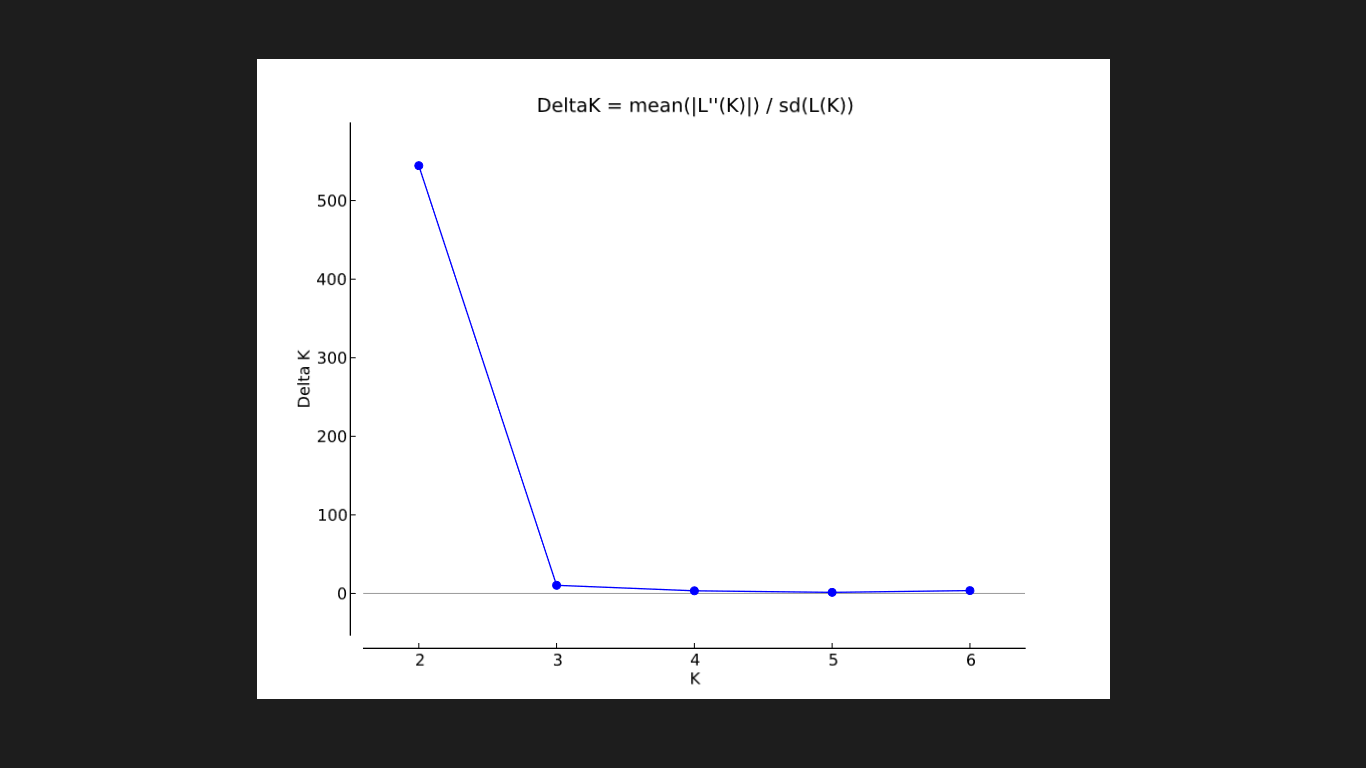


Fig S2 Rate of change in the log probability of sequential K values of wild Delmarva Peninsula sika deer (Assateague, Dorchester), captive Delmarva Peninsula sika deer (Delaware) and the source of wild sika deer (Yakushima Japan) sika deer samples as measured from 10 loci. We ran Bayesian clustering Markov Chain Monte Carlos (MCMC’s) with a burn-in of 100,000 each followed by 1,000,000 iterations. We estimated clusters (K) from 1 through 7 (n+3) with 8 iterations of each cluster. Population cluster assignment was estimated according to a slight modification of the Evanno et al. (2005) method in STRUCTURE Harvester (Earl and vonHoldt 2012).
